# The SNP rs7865618 of 9p21.3 locus emerges as the most promising marker of coronary artery disease in the southern Indian population

**DOI:** 10.1038/s41598-020-77080-4

**Published:** 2020-12-09

**Authors:** Gorre Manjula, Rayabarapu Pranavchand, Irgam Kumuda, B. Sriteja Reddy, Battini Mohan Reddy

**Affiliations:** 1 Department of Genetics, Osmania University, Hyderabad, India; 2 Molecular Anthropology Group, Indian Statistical Institute, Hyderabad, India; 3 Dr Pinnamaneni, Siddhartha Institute of Medical Sciences and Research Foundation, Vijayawada, India; 4 Emeritus Scientist (ICMR), Department of Genetics, Osmania University, Hyderabad, 500007 India

**Keywords:** Genetics, Genetic markers

## Abstract

Development of coronary artery disease (CAD) is primarily due to the process of atherosclerosis, however the prognosis of CAD depends on pleiotropic effects of the genes located at 9p21.3 region. Genome wide association studies revealed association of variants in this region with CAD pathology. However, specific marker in predicting CAD development or progression is not yet identified. In the present study, 35 SNPs at 9p21.3 region, located in the cyclin dependent kinase inhibitor (CDKN2A/CDKN2B) genes, were genotyped among 350 CAD cases and 480 controls from the southern Indian population of Hyderabad using fluidigm nanofluidic SNP genotyping system and the data were analyzed using PLINK and R softwares. Of the 35 SNPs analysed, only one SNP, rs7865618, was found to be highly significantly associated with CAD, even after correction for multiple testing (*p* = 0.008). The AG and GG genotypes of this SNP conferred 3.08 and 1.93 folds increased risk for CAD respectively. In particular, this SNP was significantly associated with severe anatomic (triple vessel disease *p* = 0.023) and phenotypic (acute coronary syndrome *p* = 0.007) categories of CAD. Pair wise SNP interaction analysis between the SNPs of 9p21.3 and 11q23.3 regions revealed significantly increased risk of three SNPs of 11q23.3 region that were not associated individually, in conjunction with rs7865618 of 9p21.3.

## Introduction

Coronary artery disease (CAD) is the most prevalent condition among cardio vascular diseases (CVD). It is characterized by the pathogenic process referred to as atherosclerosis which results in progressive damage of coronary arteries. The CAD, which is also called as coronary heart disease (CHD), clinically presents a broad range of phenotypes from less severe form—angina pectoris to most severe forms such as myocardial infarction, and/or silent myocardial ischemia^1^. Anatomically, atherosclerosis may occur in one or multiple coronary blood vessels with no clinical correlation towards the phenotypic severity. The process of atherosclerosis is primarily triggered by endothelial injury in coronary arteries due to lipoprotein metabolism abnormality or increased oxidized low density lipoproteins. Subsequent immune and inflammatory response results in disease progression until thrombosis or formation of blood clot in the lumen of the artery^2^. Therefore, it is necessary to gauge the role of genes with pleiotropic effects and involved in lipoprotein, immunity, inflammation and other metabolic processes of atherosclerosis. Genome-wide association studies (GWAS) during the past 10 years identified 163 genetic loci with risk for CAD, providing novel insights into the disease pathology^3^. Approximately half of the CAD associated loci were found to exhibit pleiotropy through their association with one or more of the CAD risk factors such as lipid traits, body mass index, diabetes and other diseases/traits^4–6^. A comprehensive literature review on the genetic etiology of CAD found 11q23.3 and 9p21.3 as the most replicated and significantly associated loci.

There is an increase in prevalence of CAD in India, which could be attributed to their characteristic low High Density Lipoproteins (HDLs) and elevated triglycerides, termed as atherogenic dyslipidaemia^7,8^. Given this, we have recently investigated the pattern of association of 95 prioritized SNPs of the GWAS identified SNPs in the 11q23.3 region^9,10^ in the CAD patients from Hyderabad, India^11,12^ and observed significant association of 12 SNPs in the intronic regions of APOA1, BUD13, ZPR1 and the intergenic region of APOA5-APOA4 genes with distinct effects towards CAD and dyslipidemia. These studies also provided insights into the nature of association of these variants with different clinical and phenotypic categories of CAD suggesting complex nature of interactions between the variants in contributing to the clinical and phenotypic heterogeneity of CAD. However, studies conducted to explain the genetic etiology of progression of atherosclerosis involving inflammation, cell cycle regulation and apoptosis are inadequate in the Indian context. Harbouring cell cycle regulating cyclin-dependent kinase inhibitor2A and cyclin-dependent kinase inhibitor2B (CDKN2A and CDKN2B) genes, 9p21.3 region was found to play a central role in cell-cycle arrest, affecting cell renewal, senescence, apoptosis and several other cellular processes^13,14^. Of the 32 CAD associated loci established from the independent GWAS and meta-analysis studies, 9p21.3 region was found to be the most replicated locus across the globe albeit lack of consistency in the association pattern was evident across different populations. The CAD associated variants of this locus were specifically found to be clustered within the 60kb intergenic region of the CDKN2A and CDKN2B-genes which is referred to as the CAD interval^15–17^. Various population based studies revealed association of different SNPs- rs10757274, rs2383206 among Caucasians and rs2383207, rs10757278 among Germans. Recently, Kalpana et al.^18^ observed rs2383206 (GG) to be associated with premature CAD among south Indians. Although this study discussed the association of several SNPs assuming various genotypic models as well as ascertained sex specific patterns, none of these SNPs were significant after Bonferroni correction for multiple testing. Further, two more variants rs7865618 (GG) and rs496892 (AA) of this locus were found associated with periodontitic coronary artery disease patients from south India^19^. However, these studies were not adequate to draw conclusions on the susceptibility profile of the concerned populations. Given the complex nature of the disease and enormous ethnic heterogeneity implicit in the Indian population, it is imperative to screen large number of populations and identify the risk associated SNPs within 9p21.3 and other CAD specific genomic regions, which can predict the development as well as prognosis of CAD.

The present study deals with a comprehensive set of 35 GWAS identified SNPs at the 9p21.3 chromosomal region for their possible association with risk for CAD and its anatomical and phenotypic sub-categories, either individually or through their interactions with other SNPs in the same region as well as with those of the 11q23.3 region.

## Results

### Allelic association of 9p21.3 SNPs with CAD

After data pruning, seven of the 35 SNPs were excluded either because of their minor allele frequency < 1% or departure from Hardy Weinberg Equilibrium (*p* < 0.001) and the remaining 28 SNPs were subjected to further analysis. The results of the logistic regression analysis of the allelic data (Table 1) suggested only one of the 28 SNPs (rs7865618) as significantly associated with CAD (*p* = 0.0003). The minor allele (G) frequency of this SNP is observed to be higher in cases (0.50) than controls (0.41), suggesting its risk conferring nature. The association remained highly significant even after correction for multiple testing (p_corrected_ = 0.008). Three more SNPs-rs1333048, rs10757274 and rs10757278 that were not significant earlier turned out to be significant (*p* ≤ 0.05) after adjusting for age and sex as covariates. However, the minor allele frequency of these SNPs was observed to be higher among the controls suggesting protective nature. On the other hand, the genotype–phenotype association analysis of rs7865618 suggested highly significant association with CAD (*p* = 3.86e^−10^), under codominant model (Table 2). Further, the AG and GG genotypes were both significantly associated and conferred 3.08 and 1.93 folds increased risk for CAD, respectively. Further, in order to test the possible association of this SNP with the common risk factors of CAD, we performed logistic regression analysis of this SNP with hypertension, diabetes and dyslipidemia, in the control cohort of this study, representing relatively large proportion (~ 38% of 500) of individuals with one of these risk factors, and observed significant risk conferring nature of association with hypertension [OR = 1.34 (1.04–1.73), *p* = 0.025] but not with either diabetes [OR = 1.25 (0.96–1.62), *p* = 0.095] or dyslipidemia [OR = 1.11 (0.86–1.43), *p* = 0.426].

**Table 1:** Allelic association of SNP variants at 9p21.3 chromosomal region with CAD.

SNP rs ID	Alleles	Minor allele frequency		χ^2^	Unadjusted		Adjusted for age, sex	
	Minor/major	CASES (N = 350)	CONTROLS (N = 480)		OR (CI 95%)	*p* value	OR (CI 95%)	*p* value
*rs7865618	G/A	0.500	0.409	13.00	1.44 (1.18–1.76)	0.0003*	1.48 (1.20–1.83)	0.0003*
rs10757274	A/G	0.435	0.477	2.939	0.84 (0.69–1.02)	0.086	0.81 (0.66–0.99)	0.040
rs10757278	A/G	0.429	0.467	2.368	0.86 (0.70–1.04)	0.124	0.81 (0.66–0.99)	0.041
rs1333048	A/C	0.414	0.458	3.205	0.84 (0.68–1.02)	0.073	0.80 (0.65–0.98)	0.036

*SNP significant after multiple correction (p_corrected = 0.008). Bold indicates the significant *p* value.

**Table 2:** Genotypic association of variants at 9p21.3 chromosomal region with CAD.

SNP	Model	Genotype	Frequency		Unadjusted	
			CASES	CONTROLS	OR (CI 95%)	*p* value
*rs7865618	Codominant	AA	0.180	0.374	Reference	**3.86e**^**10**^
		**AG**	**0.640**	0.432	**3.08 (2.18–4.37)**	
		GG	0.180	0.194	1.93 (1.25–2.99)	
log-Additive *p* value **2.07e**^**4**^						

*SNP significant for the allelic association after multiple corrections.  Bold indicates the details of significant genotype.

### Allelic association of 9p21.3 SNPs with anatomical and phenotypic categories of CAD

The results of allelic association analysis with reference to four anatomical categories of CAD viz. Insignificant, single vessel disease (SVD), double vessel disease (DVD) and, triple vessel disease (TVD) were furnished in Table 3. Except for DVD, rs7865618 is associated with increased risk for all the anatomical categories of CAD. On the other hand, the two SNPs-rs1333040 and rs10116277-conferred increased risk towards DVD. Two more SNPs, rs17694493 and rs1333048, were associated with increased and decreased risk, respectively, towards SVD. The logistic regression analyses of the SNPs with respect to phenotypic categories (Table 4) revealed highly significant association of rs7865618 with acute coronary syndrome (ACS) (*p* = 0.007) which represents the severe phenotypic form of CAD.

**Table 3:** Association of variants at 9p21.3 chromosomal region with anatomical categories of CAD.

SNP (major/minor allele)	MAF in Controls N = 480	Insignificant (n = 82)			Single vessel disease (n = 100)			Double vessel disease (n = 73)			Triple vessel disease (n = 66)		
		MAF	OR (95%CI)	*p* value	MAF	OR (95%CI)	*p* value	MAF	OR (95%CI)	*p* value	MAF	OR (95%CI)	*p* value
rs1333048(C/A)	0.458				0.373	0.70 (0.51–0.97)	0.032						
**rs17694493 (C/G)**	0.028				**0.068**	**2.52 (1.28–4.98)**	**0.006**						
*rs7865618 (A/G)	0.409	**0.493**	**1.425 (1.02–1.99)**	**0.037**	**0.500**	**1.44 (1.05–1.98)**	**0.022**				**0.516**	**1.53 (1.06–2.22)**	**0.023**
**rs1333040 (T/C)**	0.316							**0.406**	**1.48 (1.02–2.13)**	**0.036**			
**rs10116277 (T/G)**	0.338							**0.431**	**1.48 (1.02–2.15)**	**0.037**			

*Significantly associated in the sample of pooled CAD cases. Bold indicates the details of significant risk confering SNPs.

**Table 4:** Association of variants at 9p21.3 chromosomal region with stable/unstable angina, ACS, and MI categories.

SNP (major/minor allele)	MAF in controls N = 480	Angina(n = 73)			Acute coronary syndrome (n = 159)			Myocardial infarction (n = 75)		
		MAF	OR (95% CI)	*p* value	MAF	OR (95% CI)	*p* value	MAF	OR (95% CI)	*p* value
*rs7865618 (A/G)	0.409	0.486	1.36 (0.95–1.94)	0.088	**0.497**	**1.42 (1.10–1.84)**	**0.007**	0.473	1.29 (0.91–1.83)	0.151

*Significantly associated in the sample of pooled CAD cases. Bold indicates the details of significant risk confering SNP.

### Pair wise epistatic interactions of 9p21.3 SNPs and their association with CAD

The analysis of epistatic effects using pair wise logistic regression revealed significant SNP-SNP interactions of the three SNP pairs that were associated with CAD (Table 5). Interestingly, while rs7865618 is common to all the three pairs, the three other SNPs pairing with this SNP become significant only as an epistasis outcome with this highly significant variant indicating that this SNP might contribute to the pathogenesis of CAD through its interaction with other SNPs as well. However, the outcomes of these epistatic interactions are observed to be protective in nature. The haplotype and GMDR analyses for interaction among multiple SNP combinations did not yield any significant results, hence not presented.

**Table 5:** Significant SNP-SNP interaction effects on CAD obtained through pair wise logistic regression.

SNP pair		Odds ratio	*p* value
rs615552	*rs7865618	0.358	**4.71e**^**6**^
rs564398	*rs7865618	0.361	**1.42e**^**5**^
*rs7865618	rs4977756	0.394	**7.28e**^**5**^

*SNP significant for allelic and genotypic association with CAD. Bold indicates the significant risk conferring lone SNP and the *p* values of epistasis.

### Pair wise SNP–SNP interactions of 9p21.3 and 11q23.3 regions

The process of atherosclerosis is a result of disruption in lipid metabolism and cell proliferation pathways that is evident from most significant association of variants at 11q23.3 and 9p21.3 loci respectively. There is no consensus information about interaction between the variants of these loci towards disease progression. As mentioned earlier in this article, rs7865618 of 9p21.3 locus is a standalone variant significantly associated with CAD and its phenotypic or anatomical categories at allelic level and in pairwise interaction analysis. However, the protective effect of the SNP in interaction with other SNPs of the same 9p21.3 region indicated that it may contribute to CAD risk through its interactions with variants at other loci. Given the significant effect of variants in 11q23.3 region on CAD pathology, we made an attempt to explore the nature of pair wise interactions between the 35 SNPs of 9p21.3 and 95 SNPs of 11q23.3, analysed by us in the previous study^11,12^. The results revealed three significant SNP-SNP combinations between these two chromosomal loci (Table 6), each pair represented one SNP from 11q23.3 locus (rs2187126 in the intron of BUD13, rs1263163 and rs2849165 both in the intergenic region of APOA5-APOA4 genes). Interestingly, the SNP included from 9p21.3 region in all the three combinations was found to be rs7865618. None of these three SNPs at 11q23.3 locus showed significant allelic association with CAD in the earlier study, except for the protective effect of rs2849165^11^. Nevertheless, in the pair wise SNP interaction analysis, all the three were shown to be contributing significantly to CAD risk in conjunction with rs7865618 from the 9p21.3 region. Because of the absence of LD among these SNPs, the observed interactions may assume greater biological significance to the pathogenesis of CAD. These results underlined the significance of the lone SNP rs7865618 from 9p21.3 region either individually or in interaction with 11q23.3 SNPs for contributing to the risk of CAD.

**Table 6:** Significant SNP-SNP interactions between 9p21.3 and 11q23.3 regions with effects on CAD.

SNP pair		Odds ratio	*p* value
9p21.3 region	11q23.3 region		
*rs7865618	rs2187126(BUD13/Intronic)	3.485	**2.60e**^**5**^
*rs7865618	rs1263163 (APOA5-APOA4/Intergenic)	3.228	**1.98e**^**5**^
*rs7865618	rs2849165(APOA5-APOA4/ Intergenic)	3.291	**1.50e**^**8**^

*SNP significant for allelic and genotypic association with CAD. Bold indicates the significant risk conferring lone SNP and the *p* values of epistasis.

## Discussion

The complex nature of CAD phenotype might be the outcome of interactions of different genomic loci. Since the SNPs at 11q23.3 region harbouring apolipoprotein coding genes namely *APOA1, APOC3, APOA4*, *APOA5* and regulatory genes *BUD13, ZPR1, SIK3 *were found to be associated with defective lipid metabolism, this region was thought to play an important role in atherosclerosis and CAD risk. However, the distinct patterns of association of 11q23.3 SNPs observed with CAD and dyslipidemia in the previous study of this cohort made it imperative to screen for other CAD loci for deriving the genetic susceptibility profile of the population of Hyderabad. The next prominent locus identified was 9p21.3 which was found to have pleiotropic effects, including its role in atherosclerosis^20^. Recently, a male specific association of rs7865618 from this locus was reported among premature CAD cases of south Indians, albeit not significant after correction for multiple testing^18^. However, the present study confirms its association in the cohort of south Indians from Hyderabad. The association pattern of this SNP is also consistent with the earlier reports where AG and GG genotypes were shown to be significantly associated with increased risk of CAD^19,21,22^. Intriguingly, the rs7865618 that emerged as the only significant SNP associated in the pooled CAD cohort (*p* = 0.0003) also turned out to be the only significantly associated SNP with the insignificant (0.037), SVD (*p* = 0.022) and TVD (*p* = 0.023) anatomical categories and ACS (*p* = 0.007) phenotypic category. Hence, this could serve as an independent prognostic marker of the CAD in general.

This SNP is located in the CDKN2B-AS1 gene which encodes for a long non-coding antisense RNA transcript known as cyclin-dependent kinase 2B antisense RNA. It was presumed that disease risk caused by the SNPs at 9p21.3 act through this long non-coding RNA, which is commonly referred to as the antisense non-coding RNA in the INK4 locus (ANRIL). ANRIL was shown to control the expression of three major tumour suppressor loci within the INK4b-ARF-INK4a gene cluster^23^. Moreover, expression of ANRIL was observed to be specific to atherosclerotic tissues and it upregulates the cell proliferation^20^. A recent study in the south Indian population revealed significant association of rs7865618 with periodontitis (a key risk factor for CAD) and thereby suggested possible susceptibility to CAD predisposition^19^. Another study from Tehran observed similar pattern of association of rs7865618 with coronary heart disease^22^. It is plausible to surmise that the minor allele of rs7865618 may encode for a defective CDKN2B antisense RNA that affects the expression of corresponding CDKN2B gene, influencing the vascular tissue cell division and thereby causing the susceptibility to CAD development.

Concurrent to the association pattern of SNPs with DVD category, these SNPs were also observed to show significant association with CAD pathology in a north Indian population^24^. Therefore, given that the frequency of CAD cases diagnosed in this phase were comparatively lesser than that of the insignificant, SVD and TVD categories, the DVD may be regarded as a short phase that precedes the development of TVD, which might serve as an indicator of CAD progression. The SNP rs17694493 is intronic, relatively more significantly associated (*p* = 0.006) with SVD in the present study and was thought to be implicated in CAD pathology. Significant association of rs7865618 from this region with severe anatomic and phenotypic categories of CAD indicated the involvement of 9p21.3 region in CAD progression, albeit relatively larger sample size for the subcategories of CAD would have provided sufficient statistical power and greater degree of confidence in the inference. Further the interaction analysis between rs7865618 of 9p21.3 and SNPs rs2187126, rs1263163, rs2849165 of 11q23.3 indicated that the intronic variant (rs2187126) of *BUD13* gene and intergenic variants (rs1263163, rs2849165) of *APOA4-APOA5* genes have a profound risk towards CAD. Either individually or in interaction with other SNPs of 11q23.3 region, the variants of *BUD13* gene are reported to elevate lipid traits as well as conferring risk towards coronary disease. With its evolutionarily conserved function of forming pre m-RNA Retention and Splicing (RES) complex, *BUD13* appears to be one of the key regulating genes of 11q23.3 region. Further, the *APOA4-APOA5* genes are well known for their role in coding activating enzymes of cholesterol metabolism there by regulating cholesterol homeostasis^11^. It may be hypothesized from the findings of the present study that the variants in 11q23.3 chromosomal region harbouring apolipoprotein encoding or regulating genes might be involved in defective cholesterol homeostasis thereby resulting in increased levels of oxidised low density lipoproteins (LDLs). This is the first event in the process of atherosclerosis. Subsequently, the variants of other pathways such as immune and inflammation or cell cycle regulation might lead to an imbalance in further events of atherosclerosis such as invasion and functioning of cells of immune system, thereby leading to formation of fibrous cap in the inner lining of blood vessels and thrombosis or clot formation. The *CDKN2B-AS1* gene harboured at 9p21.3 region might play an important role in the later process of atherosclerosis. However, the invitro studies on the expression levels of these genes might help in validating the hypothesis.

In conclusion, the SNP rs7865618 of CDKN2B-AS1 gene from the 9p21.3 region could be a strong candidate to serve as a predictive marker for CAD risk. This SNP may be screened in relatively all the anatomic and/or phenotypic forms of CAD in order to determine the prognosis or severity of the disease. It might also be prudent to screen for rs7865618 in other complex diseases such as hypertension and type2 diabetes as precautionary/preventive measure for possible future development of CAD. However, since this SNP showed interactions with other SNPs at 11q23.3 locus in causing risk for CAD, 9p21.3 remains to be the most significant region with rs7865618 as the predictive marker that needs to be considered for functional evaluation. On the other hand, analysis of interactions between risk variants of various GWAS identified CAD loci would help in identification of causal variants for CAD complexity and phenotypic heterogeneity.

## Materials and methods

All methods were carried out in accordance with relevant guidelines and regulations. The study protocol was approved by the Indian Statistical Institute Review Committee for Protection of Research Risks to Humans. Written informed consent of all the participants is obtained as per the guidelines.

### Study design, sample and data collection

The present study was part of a major project on CAD carried out by the corresponding author at the Indian Statistical Institute, Hyderabad during 2011–2016. For this case–control study, a total of 1024 subjects comprising 508 CAD cases and 516 controls broadly representing the populations of the undivided Andhra Pradesh were included. The CAD cases were recruited from the CARE hospitals, Hyderabad, after their evaluation by interventional cardiologists. Patients with characteristic symptoms of stable/unstable angina pectoris along with variable degrees (generally > 40%) of stenosis in at least one of the major coronary arteries as determined through angiogram were included in the study. Cases with monogenic diseases, valvular heart disease, cardiomyopathy, renal disease, acute and chronic viral or bacterial infections, asthma, tumours or connective tissue diseases and other vascular diseases were excluded from the study. All the cases were evaluated by interventional cardiologists at the CARE Hospitals, Hyderabad, for the above mentioned criteria. The baseline characteristics of CAD patients recruited for the present study were already furnished in the previous paper^11^.

The CAD cases were categorized into the following four anatomical sub types^12^: (1) cases with 40–70% stenosis and symptomatic for CAD with characteristic atherosclerotic lesions as ‘insignificant’ disease, (2) with > 70% stenosis in any one of the major coronary blood vessel as ‘SVD’ (3) with > 70% stenosis in two major coronary blood vessels as ‘DVD’ and (4) with > 70% stenosis in three major coronary blood vessels are categorized as ‘TVD’. We also categorized the cases based on the phenotypic severity into three broad classes, (1) those with characteristic symptoms of stable or unstable angina, (2) with symptoms of ACS, and (3) with reported myocardial infarction (MI). We could not retrieve relevant information for categorizing CAD cases into the above subcategories for some of the case samples, hence there was a difference in the total number of CAD cases used for anatomical and phenotypic severity categories when compared to the sample of pooled CAD cases.

The control samples were collected from Hyderabad and its vicinity, broadly representing similar ethnic composition, socioeconomic backgrounds as that of the cases, and aged above 45 years. The population of Hyderabad is a conglomeration of people from different parts of the undivided state of Andhra Pradesh and the mother tongue of most of its population is Telugu, one of the four Dravidian languages. It would be also pertinent to note that despite the subdivision of Telugu population into a number of traditionally endogamous castes and sub castes, Reddy et al.^25^ observed genetic differentiation among the populations of Andhra Pradesh to be very low and insignificant; the Markov chain Monte Carlo analysis of population structure, which implements model based clustering method for grouping individuals into populations^26,27^, did not reveal any unique population clusters, suggesting high degree of genetic homogeneity.

The epidemiological and clinical data pertaining to the individuals who participated in the study were obtained through personal interviews using a detailed questionnaire, and from the hospital records. About 5 ml of peripheral fasting blood sample was collected from each of the subjects by certified medical lab technicians. All blood samples were used for isolation of DNA using phenol chloroform method^28^. The quality and quantity of isolated DNAs were determined with the help of Thermo Scientific Varioskan Flash Multimode Reader using Quant-iT PicoGreen dsDNA Assay Kit. Quantification of the samples was done at Sandor Lifesciences, a medical laboratory in Hyderabad.

### SNP selection, genotyping and statistical analysis

The 35 SNPs that were significantly associated with CAD in GWAS and in validation studies until 2016 (start of the project) were considered for genotyping. Genotyping was performed for these-SNPs using Fluidigm nanofluidic SNP genotyping system at Sandor Life Sciences, Hyderabad and the methodology was detailed in earlier publication mentioned above^11^. For the present study, we have genotyped all the 35 SNPs (Table S1) in 350 cases and 480 controls with call rate of ≥ 99% and used for association analyses. The data pruning, logistic regression analysis with and without covariates and pair-wise SNP-SNP interaction analysis were done using PLINK software version1.07. The CAD cases were also categorized into four ‘anatomical’ subtypes (insignificant, SVD, DVD and TVD), and three ‘phenotypic’ severity categories (angina, ACS and MI) for further analysis of allelic association using PLINK. The ‘SNPassoc’ package of R-PROGRAM was used for genotypic association analyses assuming different models.

### Ethics approval and consent to participate

The study protocol was approved by the Indian Statistical Institute Review Committee for Protection of Research Risks to Humans. Written informed consent of all the participants is obtained as per the guidelines.

### Consent for publication

All authors agree to publish this article in the journal of Scientific Reports.

## Supplementary information

Supplementary Table S1.

## Data Availability

The datasets used and/or analyzed during the current study are available from the corresponding author on reasonable request.

## References

[1] Cervellin G, Lippi G (2014). Of MIs and men-a historical perspective on the diagnostics of acute myocardial infarction. Semin. Thromb. Hemost..

[2] Nelson CP (2017). Association analyses based on false discovery rate implicate new loci for coronary artery disease. Nat. Genet..

[3] Jeanette E, Thorsten K, Loreto MV, Heribert S (2018). A decade of genome-wide association studies for coronary artery disease: the challenges ahead. Cardiovasc. Res..

[4] Webb TR (2017). Systematic evaluation of pleiotropy identifies 6 further loci associated with coronary artery disease. J. Am. Coll. Cardiol..

[5] Chesmore K, Bartlett J, Williams SM (2018). The ubiquity of pleiotropy in human disease. Hum. Genet..

[6] Ross R, Glomset J, Harker L (1977). Response to injury and atherogenesis. Am. J. Pathol..

[7] Sekhri T (2014). Prevalence of risk factors for coronary artery disease in an urban Indian population. BMJ Open.

[8] Joshi SR (2014). Prevalence of dyslipidemia in urban and rural India: the ICMR–INDIAB study. PLoS ONE.

[9] Braun TR (2014). A replication study of GWAS-derived lipid genes in Asian Indians: the chromosomal region 11q23.3 harbors loci contributing to triglycerides. PLoS ONE.

[10] Willer CJ (2013). Discovery and refinement of loci associated with lipid levels. Nat. Genet..

[11] Rayabarapu PC, Arramraju SK, Kapadia A, Satti V, Battini MR (2016). Distinct patterns of association of variants at 11q23.3 chromosomal region with coronary artery disease and dyslipidemia in the population of Andhra Pradesh, India. PLoS ONE.

[12] Rayabarapu PC, Arramraju SK, Battini MR (2017). Genetic determinants of clinical heterogeneity of the coronary artery disease in the population of Hyderabad, India. Hum. Genom..

[13] Gil J, Peters G (2006). Regulation of the INK4b-ARF-INK4a tumour suppressor locus: all for one or one for all. Nat. Rev. Mol. Cell Biol..

[14] Popov N, Gil J (2010). Epigenetic regulation of the INK4b-ARFINK4a locus: in sickness and in health. Epigenetics.

[15] Burd CE, Jeck WR, Liu Y, Sanoff HK, Wang Z, Sharpless NE (2010). Expression of linear and novel circular forms of an INK4/ARF-associated non-coding RNA correlates with atherosclerosis risk. PLoS Genet..

[16] Bansal V (2010). Accurate detection and genotyping of SNPs utilizing population sequencing data. Genome Res..

[17] Harismendy O (2011). 9p21 DNA variants associated with coronary artery disease impair interferon-gamma signalling response. Nature.

[18] Bellary K (2019). Genetic variants of chromosome 9p21.3 region associated with coronary artery disease and premature coronary artery disease in an Asian Indian population. Indian Heart J..

[19] Madhavi M (2019). Association of CDKN2BAS gene polymorphism with periodontitis and coronary artery disease from South Indian population. Gene.

[20] Kim JB (2012). Effect of 9p21.3 coronary artery disease locus neighboring genes on atherosclerosis in mice. Circulation.

[21] Samani NJ (2007). Genome wide association analysis of coronary artery disease. N. Engl. J. Med..

[22] Matoo S (2017). Increased risk of CHD in the presence of rs7865618 (A allele): Tehran lipid and glucose study. Arch. Iran. Med..

[23] Yu W (2008). Epigenetic silencing of tumour suppressor gene p15 by its antisense RNA. Nature.

[24] Kumar J (2011). Association of polymorphisms in 9p21 region with CAD in North Indian population: replication of SNPs identified through GWAS. Clin. Genet..

[25] Reddy BM (2005). Microsatellite diversity in Andhra Pradesh, India: genetic stratification versus social stratification. Hum. Biol..

[26] Pritchard JK, Stephens M, Donnelly P (2000). Inference of population structure using multilocus genotype data. Genetics.

[27] Falush D, Stephens M, Pritchard JK (2003). Inferences of population structure using multilocus genotype data: linked loci and correlated allele frequencies. Genetics.

[28] Sambrook J, Fritschi EF, Maniatis T (1989). Molecular Cloning: A Laboratory Manual.

