# Supplementary material for: The SNP rs7865618 of 9p21.3 locus emerges as the most promising marker of coronary artery disease in the southern Indian population
Source: Sci Rep. 2020 Dec 9;10:21511. doi: 10.1038/s41598-020-77080-4 (PMC7726101; doi:10.1038/s41598-020-77080-4)
Supplement: Supplementary file 1 — Supplementary Table S1. [file 41598_2020_77080_MOESM1_ESM.docx]

**Title of the Manuscript:**

The SNP rs7865618 of 9p21.3 locus emerges as the most promising marker of Coronary Artery Disease in the southern Indian population

**Author list:**

Gorre Manjula^1^, Rayabarapu Pranavchand^2#^, Irgam Kumuda^1^, B Sriteja Reddy^3^, Battini

Mohan Reddy Ph.D^1, 2*#^

^1^Department of Genetics, Osmania University, Hyderabad, India; ^2^Molecular Anthropology Group, Indian Statistical Institute, Hyderabad, India; ^3^Dr Pinnamaneni Siddhartha Institute of Medical Sciences & Research Foundation, Vijayawada, India

**Short title:** Association of the variants of 9p21.3 locus with CAD

**^*^*Corresponding author***

Professor B M Reddy

Emeritus Scientist (ICMR)

Department of Genetics

Osmania University

HYDERABAD – 500007, INDIA

E-mail: bmrisi@gmail.com; Mobile: 9866289771

***# Sample collection, DNA isolation and genotyping of the SNPs for this study accomplishedwhenBMRwas working as Professor of Indian Statistical Institute, and RP was an SRF associated withBMR as Ph.D student.***

***Scientific Reports, June 2020***

| **Table S1. Details, allele and genotype frequencies of 35 SNPs selected from 9p21.3 chromosomal region** | | | | | | | | | | |
| --- | --- | --- | --- | --- | --- | --- | --- | --- | --- | --- |
| **S.no.** | **SNP** | **minor**  **allele** | **maf controls (n=480)** | **maf cases (n=350)** | **odds ratio** | **p value** | **genotype frequencies**  **variant/hetero/wild (controls)** | **genotype frequencies**  **variant/hetero/wild**  **(cases)** | **chi-square value** | **p value** |
| 1 | rs7023329 | G | 0.3078 | 0.3107 | 1.014 | 0.8993 | 53/187/236 | 34/147/165 | 0.9757 | 0.6139 |
| 2 | rs3731239 | C | 0.1715 | 0.1667 | 0.9665 | 0.8029 | 33/89/330 | 18/74/238 | 1.702 | 0.427 |
| 3 | rs2811712 | G | 0.1531 | 0.1173 | 0.7351 | 0.0393 | 27/89/351 | 2/76/263 | NA | NA |
| 4 | rs3218018 | C | 0.03151 | 0.03488 | 1.111 | 0.7058 | 5/20/451 | 0/24/320 | NA | NA |
| 5 | rs3218009 | C | 0.00315 | 0.01017 | 3.252 | 0.0714 | 0/3/473 | 2/3/339 | NA | NA |
| 6 | rs3217992 | A | 0.4113 | 0.3944 | 0.9322 | 0.494 | 91/203/174 | 62/145/134 | 0.4319 | 0.8058 |
| 7 | rs1063192 | C | 0.2025 | 0.2126 | 1.063 | 0.6202 | 27/138/309 | 18/109/214 | 0.7775 | 0.6779 |
| 8 | rs615552 | G | 0.2179 | 0.2257 | 1.046 | 0.7124 | 27/150/291 | 22/109/208 | 0.1902 | 0.9093 |
| 9 | rs564398 | G | 0.2089 | 0.2133 | 1.027 | 0.8292 | 27/144/303 | 20/108/219 | 0.0585 | 0.9712 |
| 10 | rs7865618 | G | 0.4096 | 0.5 | 1.442 | 0.0003 | 91/203/176 | 61/216/61 | 41.67 | 8.93E+10 |
| 11 | rs17694493 | G | 0.0283 | 0.03913 | 1.398 | 0.2241 | 0/27/450 | 0/27/318 | NA | NA |
| 12 | rs1011970 | T | 0.2826 | 0.3052 | 1.115 | 0.3191 | 43/183/250 | 35/140/169 | 0.9803 | 0.6125 |
| 13 | rs4977756 | G | 0.1956 | 0.2038 | 1.053 | 0.6815 | 22/141/310 | 15/111/220 | 0.4972 | 0.7799 |
| 14 | rs16905599 | A | 0.2574 | 0.2805 | 1.125 | 0.2986 | 40/162/268 | 31/131/182 | 1.386 | 0.5001 |
| 15 | rs10116277 | G | 0.3376 | 0.3466 | 1.041 | 0.7066 | 56/204/208 | 48/139/152 | 1.05 | 0.5915 |
| 16 | rs10965227 | G | 0.1427 | 0.1526 | 1.082 | 0.5763 | 13/109/351 | 15/75/254 | 1.65 | 0.4382 |
| 17 | rs6475606 | C | 0.3446 | 0.3469 | 1.01 | 0.9222 | 56/214/203 | 44/150/149 | 0.273 | 0.8724 |
| 18 | rs1547705 | C | 0.03145 | 0.01308 | 0.4082 | 0.0159 | 7/16/454 | 0/9/335 | NA | NA |
| 19 | rs1333040 | C | 0.3162 | 0.3271 | 1.051 | 0.6395 | 44/213/219 | 39/149/159 | 0.9432 | 0.624 |
| 20 | rs10757272 | C | 0.3614 | 0.3574 | 0.9828 | 0.8701 | 63/205/190 | 44/150/139 | 0.04847 | 0.9761 |
| 21 | rs10757274 | A | 0.4775 | 0.4346 | 0.841 | 0.0865 | 107/232/128 | 67/165/112 | 2.983 | 0.225 |
| 22 | rs4977574 | A | 0.4668 | 0.4401 | 0.8977 | 0.2858 | 101/234/132 | 71/159/112 | 1.917 | 0.3835 |
| 23 | rs1333042 | A | 0.3468 | 0.3396 | 0.9685 | 0.7617 | 60/206/204 | 43/149/154 | 0.1004 | 0.9511 |
| 24 | rs2383206 | A | 0.4478 | 0.4099 | 0.8567 | 0.1276 | 95/230/144 | 61/160/123 | 2.465 | 0.2915 |
| 25 | rs2383207 | A | 0.334 | 0.325 | 0.9599 | 0.7023 | 56/204/213 | 38/145/157 | 0.1428 | 0.9311 |
| 26 | rs1333045 | T | 0.45 | 0.4277 | 0.9135 | 0.3733 | 97/229/144 | 67/156/116 | 1.163 | 0.5592 |
| 27 | rs10757278 | A | 0.467 | 0.4286 | 0.8559 | 0.1238 | 101/237/132 | 67/160/116 | 3.084 | 0.2139 |
| 28 | rs1333048 | A | 0.4584 | 0.4137 | 0.8338 | 0.0734 | 94/242/133 | 63/157/122 | 4.937 | 0.08473 |
| 29 | rs1333049 | G | 0.4789 | 0.442 | 0.8622 | 0.1401 | 103/247/123 | 71/163/111 | 3.773 | 0.1516 |
| 30 | rs2891169 | A | 0.4841 | 0.4677 | 0.9365 | 0.5144 | 106/245/121 | 79/161/101 | 2.067 | 0.3557 |
| 31 | rs2383208 | G | 0.1406 | 0.1541 | 1.113 | 0.4465 | 11/111/351 | 9/88/247 | 0.5916 | 0.7439 |
| 32 | rs10811661 | C | 0.1899 | 0.1971 | 1.047 | 0.7143 | 55/70/349 | 35/66/244 | 2.907 | 0.2337 |
| 33 | rs10757283 | T | 0.4788 | 0.4912 | 1.051 | 0.6217 | 106/239/126 | 92/150/98 | 3.791 | 0.1502 |
| 34 | rs7853123 | A | 0.2947 | 0.2831 | 0.945 | 0.6181 | 60/147/246 | 43/98/184 | 0.4998 | 0.7789 |
| 35 | rs1808325 | A | 0.46 | 0.4667 | 1.027 | 0.7892 | 103/231/141 | 86/150/109 | 2.293 | 0.3177 |
